# Preoperative Bioelectrical Impedance Analysis Identifies Altered Body Composition, Which Is Associated with Severe Postoperative Complications in Major Hepatopancreaticobiliary Surgery

**DOI:** 10.3390/diagnostics16172792

**Published:** 2026-08-30

**Authors:** Clemens Schmutzhart, Martin Varga, Julian Seethaler, Tarkan Jäger, Gunnar Treff, Giorjines Boppre, Jan David Smeddinck, Josef Niebauer, Wolfgang Hitzl, Klaus Emmanuel, Stefan Löb

**Affiliations:** 1 Department of Surgery, Paracelsus Medical University, University Hospital Salzburg, 5020 Salzburg, Austria; m.varga@salk.at (M.V.); j.seethaler@salk.at (J.S.); ta.jaeger@salk.at (T.J.); k.emmanuel@salk.at (K.E.); s.loeb@salk.at (S.L.); 2 University Institute of Sports Medicine, Prevention and Rehabilitation, Paracelsus Medical University, University Hospital Salzburg, 5020 Salzburg, Austria; gunnar.treff@pmu.ac.at (G.T.); giorjines.boppre@pmu.ac.at (G.B.); j.niebauer@salk.at (J.N.); 3 Ludwig Boltzmann Institute for Digital Health and Prevention, 5020 Salzburg, Austria; jan.smeddinck@dhp.lbg.ac.at; 4 Department of Ophthalmology and Optometry, Paracelsus Medical University, University Hospital Salzburg, 5020 Salzburg, Austria; w.hitzl@salk.at

**Keywords:** hepatopancreaticobiliary surgery, bioelectrical impedance analysis, postoperative complications, performance status, altered body composition

## Abstract

**Background:** Altered body composition is common in patients undergoing major hepatopancreaticobiliary (HPB) surgery and may be associated with increased postoperative morbidity. Conventional markers such as body mass index (BMI) and serum albumin have limited sensitivity for detecting early nutritional changes. Bioelectrical impedance analysis (BIA) provides an objective assessment of body composition, and phase angle (PA) may serve as a proxy for cellular status. **Methods:** This single-center retrospective observational cohort study included 73 patients undergoing major HPB surgery. Preoperative BIA-derived variables included resistance (R), reactance (Xc), PA, fat-free mass index (FFMI), fat mass index (FMI), and the Body Composition Chart (BCC). Nutritional Risk Screening 2002 (NRS 2002), serum albumin, and prealbumin were assessed preoperatively. BIA-derived data were compared with published healthy reference data. Postoperative complications were graded according to the Clavien–Dindo classification. **Results:** Patients demonstrated significant alterations in BIA-derived variables compared with healthy controls. Xc and PA were significantly reduced across all analyzable/matched sex and BMI strata (all *p* < 0.05). The median PA in the surgical cohort was 4.3° and significantly lower than reference values for both female and male patients (*p* < 0.001). FFMI and FMI distributions indicated a high prevalence of adverse body composition phenotypes, including low fat-free mass and increased adiposity. In multivariable Firth penalized logistic regression, a BCC classification outside the green reference zone (odds ratio (OR) 3.32, 95% confidence interval (CI) 1.04–12.87, *p* = 0.043) and higher Eastern Cooperative Oncology Group (ECOG) performance status (OR 2.07, 95% CI 1.08–4.24, *p* = 0.028) were independently associated with severe postoperative complications. **Conclusions:** Patients undergoing major HPB surgery frequently present with relevant preoperative nutritional and cellular impairments that are not adequately identified by BMI and serum albumin. Incorporation of BIA into routine preoperative assessment may improve risk stratification and support targeted nutritional optimization in this high-risk surgical population.

## 1. Introduction

The worldwide incidence of gastrointestinal malignancies is rising [1]. Complete surgical resection of HPB tumors remains the cornerstone of therapy and is associated with long-term survival or even cure. However, in patients undergoing major hepatopancreatobiliary (HPB) surgery, reported postoperative morbidity rates vary between 30 and 60 percent. The average postoperative mortality rate is reported to be between 5 and 20 percent [2].

Malnutrition is a widespread and serious concern among individuals with hepatobiliary and pancreatic tumors. These patients often experience considerable body mass loss and cachexia, primarily due to tumor-related insufficient nutritional intake, hypermetabolism, and inflammation. Furthermore, poor nutritional status in patients with cancer is closely linked to an increased risk of postoperative complications, a prolonged length of hospital stay, and postoperative mortality [3]. Consequently, early identification of malnutrition in the preoperative period is crucial, as physical and nutritional prehabilitation strategies have been shown to improve surgical outcomes [3,4,5].

Given the clinical relevance of malnutrition in this population, accurate assessment of nutritional status is essential. Traditional nutritional markers, such as serum albumin, can be misleading because albumin is affected by many factors, such as intravascular fluid status and inflammation. Consequently, preoperative albumin levels may not accurately reflect a patient’s nutritional status [3,6,7,8].

Prealbumin has a shorter half-life, is less affected by inflammation and thus a more sensitive marker of malnutrition than albumin. A prealbumin level below 20 mg/dL is used as a cutoff for malnutrition and has been associated with higher postoperative infection rates [3,9,10]. Hence, relying solely on preoperative albumin levels may result in overlooking many at-risk patients. As a result, preoperative nutritional assessments based solely on conventional biochemical markers may be insufficient in this population, requiring additional nutritional evaluation methods [3,5,6,11].

BIA is a noninvasive method for assessing body composition, enabling a differentiated evaluation of body compartments and hydration status. It is based on the principle that tissues differ in their electrical conductivity: water- and electrolyte-rich tissues (such as muscle) conduct electrical current well, whereas adipose tissue shows much lower conductivity. These differences allow BIA to distinguish between various body compartments. In addition, specific BIA-derived variables may provide surrogate indicators related to cellular integrity [6]. Specifically, BIA derives this information by measuring the tissue resistance (R) and reactance (Xc) to a small electrical current, enabling the assessment of body composition (BC), estimating fat mass, muscle mass, as well as fluid compartments. The phase angle (PA) derived from R and Xc reflects cellular membrane integrity and body cell mass. Higher PA values indicate robust cell mass and membrane function, whereas lower PA values suggest cell membrane breakdown and reduced lean body mass, which are commonly interpreted as markers of malnutrition or frailty. In addition, the fat-free mass index (FFMI) quantifies metabolically active tissue, while the fat mass index (FMI) represents total fat mass and allows for a more differentiated assessment of adiposity than body mass index (BMI) alone [12,13].

Therefore, FFMI and FMI help identify nutritional phenotypes, such as altered body composition or reduced fat-free mass, both of which are associated with an increased postoperative complication rate [3,5,11]. While computed tomography (CT) and magnetic resonance imaging (MRI) remain the gold standards for the precise anatomical quantification of skeletal muscle mass, calf circumference, and handgrip strength are the recommended clinical tools for assessing muscle function, these methods are not always routinely available or feasible for rapid bedside functional assessment. BIA aims to complement these established modalities by providing immediate, non-invasive insights into fluid distribution and cellular integrity [14,15,16].

Beyond BC, evidence across surgical disciplines has identified a low PA as an objective prognostic marker of poor postoperative outcomes [3,12,17].

In a systematic review of patients who underwent cancer surgery, 8 of 12 studies reported significant associations between BIA-derived parameters and postoperative complications, with phase angle consistently outperforming other BIA-based measures. Similarly, studies on major abdominal surgery have shown that preoperative PA values below ~5° are associated with higher morbidity, with low PA values identified as an independent predictor of postoperative complications. Collectively, these findings underscore the clinical relevance of BIA-derived markers of nutritional status as powerful indicators of surgical risk [3].

To our knowledge, only limited data have evaluated comprehensive preoperative BIA—including bioelectrical impedance vector analysis (BIVA) and Body Composition Chart (BCC) analysis—in a contemporary cohort undergoing major HPB surgery while simultaneously integrating nutritional risk screening, biochemical markers, and postoperative outcomes. Therefore, this study aimed to evaluate whether standardized BIA can practically complement established screening methods to identify high-risk phenotypes in routine clinical care. We hypothesized that patients with HPB malignancies would present with an abnormal impedance vector (characterized by higher R and lower Xc) indicative of cellular impairment compared with an age- and BMI-matched healthy reference population, and that these objective BIA-derived parameters would be independently associated with severe postoperative morbidity [14].

## 2. Methods

### 2.1. Study Design and Setting

This is a single-center retrospective observational cohort study conducted at the Department of Surgery, University Clinic of Salzburg, between January 2024 and October 2025. The study cohort comprised patients undergoing major pancreatic or hepatic resections, including pancreatoduodenectomy, distal pancreatectomy, hemihepatectomy, or multiple segmentectomies. In selected cases, combined hepato-pancreatic procedures were performed, such as synchronous liver metastasectomy during pancreatic surgery or hepatic tumor resection requiring en bloc pancreatoduodenectomy in cases of extended bile duct cancer.

Patient demographic and perioperative data, including age, sex, and ethnicity, as well as perioperative data, were obtained from the medical records. Nutritional risk screening was performed according to the guidelines of the European Society for Clinical Nutrition and Metabolism (ESPEN) [7]. As part of the preoperative assessment, all patients underwent BIA assessments before surgery.

### 2.2. Bioelectrical Impedance Analysis and Laboratory Measurements

Whole-body measurements were performed in the standing position using a phase-sensitive, multi-frequency, eight-electrode device (seca mBCA-Ultra; seca GmbH & Co. KG, Hamburg, Germany). The primary raw impedance parameters collected were resistance (R) and reactance (Xc) at 50 kHz, from which the phase angle (PA) was calculated using the SECA software. To account for differences in body size, impedance data were normalized to body height. Fat-free mass index (FFMI) and fat mass index (FMI) were calculated accordingly.

To evaluate body composition phenotypes, these indices were plotted on a body composition chart (BCC), allowing for a visual categorization of the subject’s functional status relative to healthy reference populations. Additionally, a qualitative, equation-independent assessment of hydration status and cell mass integrity was performed using bioelectrical impedance vector analysis (BIVA). For BIVA, height-normalized resistance (R/H) and reactance (Xc/H) are plotted as a single vector on an RXc point graph and evaluated against population-specific tolerance ellipses [6,18]. Standard laboratory tests and nutritional serum markers (albumin and prealbumin) were collected.

### 2.3. Nutritional Risk Screening

Preoperative nutritional risk was evaluated using the Nutritional Risk Screening (NRS 2002) tool in accordance with the guidelines of the European Society for Clinical Nutrition and Metabolism. This validated screening process consists of an initial phase followed by a comprehensive final assessment, resulting in a total score ranging from 0 to 7. The final score is calculated as the cumulative sum of a nutritional score (0–3), which evaluates the patient’s status based on body mass index, weight loss, and recent dietary intake, and a severity of disease score (0–3) that reflects the increased metabolic requirements caused by the underlying condition, such as major abdominal surgery. Furthermore, an age adjustment is performed by adding an additional point for patients aged 70 years or older to account for reduced physiological reserve. A total score of 3 or higher identifies the patient as being at nutritional risk, necessitating the implementation of a targeted nutritional care plan [7].

### 2.4. Outcomes

Preoperative nutritional status was evaluated using BIA-derived variables, including FFMI, FMI, R, Xc, and PA. These were analyzed to characterize alterations in BC, hydration status, and cellular health in patients undergoing major HPB surgery and were compared with age- and BMI-matched healthy reference data.

Postoperative outcomes were assessed to examine their relationship with preoperative nutritional status. These included postoperative complications according to the Clavien-Dindo score, length of stay (LOS), in-house mortality rate, and readmission rate within 30 days [19].

### 2.5. Reference Population

Control data were obtained from two independent databases. First, normative percentiles for the FFMI and FMI were derived from a cohort of 1050 healthy adults reported by Peine et al. Second, reference values for R, Xc, and PA were obtained from Piccoli et al. [6,18].

### 2.6. Statistical Analysis

Descriptive data are presented as means with standard deviations (±SD) for normally distributed variables and as medians with interquartile ranges (IQR) for non-normally distributed variables. Categorical variables are reported as frequencies and percentages (%). Analyses were performed stratified by sex and BMI categories, as appropriate. Comparisons of continuous BIA-derived variables within the study cohort across BMI categories were conducted using Welch’s *t*-test for normally distributed variables or the Mann–Whitney U test for non-normally distributed variables. Comparisons between the surgical cohort and the age- and BMI-matched healthy reference populations were performed using Welch’s *t*-test when reference group summary statistics were available. In cases where individual-level reference data were not available, Glass’s Δ was used as an approximate measure of effect size, with the standard deviation of the reference population serving as the standardizer. A cut-off threshold of ±0.5 was applied to indicate a clinically relevant deviation. Categorical postoperative outcomes, including Clavien–Dindo grade ≥ 3 complications, were compared between groups using the Chi-square test or Fisher’s exact test, as appropriate. For these comparisons, odds ratios (ORs) and risk differences (RDs) with 95% confidence intervals (CIs) were calculated.

Spearman’s rank correlation coefficients were calculated to evaluate the strength and direction of monotonic associations between continuous or ordinal variables, such as BIA-derived parameters, laboratory markers, and clinical scores. Furthermore, regression analyses were performed to evaluate factors independently associated with the respective outcomes while adjusting for potential confounding factors. Linear regression models were used for continuous dependent variables, whereas multivariable logistic regression models were applied for binary outcomes (e.g., occurrence of complications), reporting adjusted odds ratios (ORs) with corresponding 95% CIs.

To evaluate factors associated with severe postoperative complications (Clavien–Dindo grade ≥ 3) while limiting the risk of statistical overfitting, a predefined set of four clinically relevant variables was selected: “BCC out of green”, ECOG performance status, ASA physical status, and age. Each variable was first evaluated separately using univariate Firth penalized logistic regression and was subsequently included jointly in the multivariable Firth penalized logistic regression model. The ASA and ECOG scores were selected because they summarize the overall burden of comorbidity and functional impairment without requiring the inclusion of multiple individual disease variables. Odds ratios (ORs) with corresponding 95% confidence intervals (CIs) were reported.

All statistical tests were two-sided, and a *p*-value < 0.05 was considered statistically significant. Statistical analyses were performed using Python version 3.14.5 (Python Software Foundation, Wilmington, DE, USA) and R software version 4.6.0 (R Foundation for Statistical Computing, Vienna, Austria).

During the preparation of this manuscript, generative AI (Google Gemini) was used as an author support tool. Specifically, it assisted with English language polishing, grammar correction, formatting, and data consistency checks between the main text and Supplementary Materials. It was also used to assist in drafting the submission cover letter. All AI-generated suggestions were critically reviewed, validated, and edited by the human authors. The authors maintain full oversight and take full responsibility for the accuracy, originality, and integrity of the final submitted manuscript.

## 3. Results

A total of 73 patients were included in the analysis (Table 1). The median age was 70 years (IQR 61–78) and 26 patients (36%) were female. The mean body height was 170.5 cm, with a standard deviation of 8.8 cm.

The median body mass index was 25.1 kg/m^2^ (IQR 22.6–27.4). With regard to preoperative physical status, three patients (4%) were classified as ASA 1, 13 patients (18%) as ASA 2 and 57 patients (78%) as ASA 3. ECOG performance status was 0 in 30 patients (41%), 1 in 31 (42%), 2 in 10 (14%), and 3 in 2 (3%). The nutritional risk score (NRS) showed a median of 2 (IQR 1–4). Bioelectrical impedance analysis revealed a median R (resistance) of 541.6 Ω (IQR 501.0–623.2), a median Xc (reactance) of 41.5 Ω (IQR 34.8–49.9), and a median PA (phase angle) of 4.3 (IQR 3.6–4.8). The FFMI (fat-free mass index) was 18.5 kg/m^2^ (IQR 15.5–20.1), and the FMI (fat mass index) was 8.5 kg/m^2^ (IQR 6.6–10.9). For hematological variables, the median hemoglobin concentration was 12.5 g/dL (IQR 11.4–13.6), the median serum albumin level was 4.0 g/dL (IQR 3.7–4.3) and the median prealbumin concentration was 19.8 mg/dL (IQR 14.9–24.8).

Regarding surgical interventions, 45 patients (62%) underwent pancreaticoduodenectomy, 10 patients (14%) underwent distal pancreatectomy, 5 patients (7%) underwent total pancreatectomy, 5 patients (7%) underwent right hemihepatectomy, and 8 patients (10%) underwent multisegmentectomy or other procedures. Postoperatively, 27 patients (37%) experienced complications classified as Clavien–Dindo grade ≥ 3. The 30-day mortality rate was 7% (*n* = 5). The median LOS was 15 days, with an IQR of 11 days to 28 days. The readmission rate within 30 days was 5% (*n* = 4).

The 27 severe complications comprised 13 Clavien–Dindo grade 3 events, 9 grade 4 events, and 5 grade 5 events. These severe complications encompassed a spectrum of typical major surgical morbidities, including intraabdominal retentions requiring endoscopic intervention (*n* = 5, 7%), Grade B/C postoperative pancreatic fistulas (*n* = 5, 7%), abdominal dehiscence and major surgical site infections (*n* = 3, 4%), anastomotic insufficiencies (*n* = 4, 6%) and bleeding events (*n* = 4, 6%) requiring operative intervention, as well as fatal cardiovascular ischemic events (*n* = 3, 4%) and systemic organ failure or unspecified causes (*n* = 2, 3%) (Table 2).

### 3.1. Resistance

In female patients, the mean R normalized to body height (Ω/cm) was 3.76 ± 0.74 across the entire cohort. When stratified by BMI, the R/body height was 3.98 ± 0.71 Ω/cm for those with a BMI < 25, 3.50 ± 0.49 Ω/cm for individuals with a BMI ranging from ≥25 to <30, and 2.79 ± 0.99 Ω/cm for those with a BMI ≥ 30. Among male patients, the overall mean R/body height was 3.07 ± 0.57 Ω/cm, with specific values of 3.38 ± 0.67 Ω/cm for a BMI < 25, 2.87 ± 0.36 Ω/cm for a BMI between ≥25 and <30, and 2.78 ± 0.39 Ω/cm for a BMI ≥ 30 (Table 3).

### 3.2. Reactance

In the analysis of Xc normalized to body height (Ω/cm), female participants exhibited a mean value of 0.26 ± 0.07 Ω/cm overall. Specifically, the reactance/height was 0.27 ± 0.07 Ω/cm in the group with a BMI < 25, 0.24 ± 0.08 Ω/cm in the BMI ≥ 25 to <30 group, and 0.23 ± 0.11 Ω/cm in individuals with a BMI ≥ 30. Correspondingly, male participants demonstrated an overall mean value of 0.24 ± 0.06 Ω/cm, with values of 0.25 ± 0.06 Ω/cm for BMI < 25, 0.23 ± 0.05 for BMI ≥ 25 to <30, and 0.22 ± 0.07 Ω/cm for BMI ≥ 30 (Table 3).

### 3.3. Phase Angle

Regarding the phase angle (PA) (°), female patients demonstrated an overall arithmetic mean of 3.85 ± 0.87°. Specifically, the phase angles were 3.78 ± 0.88° for patients with a BMI < 25, 3.83 ± 0.94° for those with a BMI ≥ 25 to <30, and 4.50 ± 0.71° for patients with a BMI ≥ 30. Among male patients, the overall mean phase angle was 4.41 ± 0.73°, with values of 4.28 ± 0.67°, 4.55 ± 0.77°, and 4.32 ± 0.75° corresponding to BMI < 25, BMI ≥ 25 to <30, and BMI ≥ 30, respectively (Table 3).

### 3.4. Fat-Free Mass Index

For the of the fat-free mass index (FFMI, kg/m^2^) analysis, female patients exhibited a mean value of 14.42 ± 1.56 kg/m^2^ within the BMI < 25 category. This index increased to 17.33 ± 2.20 kg/m^2^ among those with a BMI ranging from ≥25 to <30 and further rose to 23.85 ± 5.30 kg/m^2^ in individuals with a BMI ≥ 30. Conversely, in male patients, the mean FFMI was 17.15 ± 2.13 kg/m^2^ for those with a BMI < 25, increased to 20.05 ± 2.28 kg/m^2^ in the BMI ≥ 25 to <30 group, and reached 21.55 ± 2.34 kg/m^2^ in patients with a BMI ≥ 30 (Table 3).

### 3.5. Fat Mass Index

For FMI kg/m^2^ analysis, female patients exhibited a mean FMI of 7.31 ± 1.96 kg/m^2^ within the BMI < 25 category. This value increased to 14.91 ± 10.14 kg/m^2^ among those with a BMI ranging from ≥25 to <30 and further rose to 17.45 ± 8.13 kg/m^2^ in individuals with a BMI ≥ 30. Conversely, male patients presented FMI values of 6.37 ± 1.86 kg/m^2^ for BMI < 25, 8.22 ± 2.20 kg/m^2^ for BMI ≥ 25 to <30, and 12.98 ± 1.97 kg/m^2^ for those with a BMI ≥ 30 (Table 3).

### 3.6. Comparative Analysis

For the comparative analysis of resistance, reactance and phase angle, six female study participants with a BMI ≥ 30 were excluded due to missing corresponding matched controls within the healthy population (Table 4, Supplementary Figure S1, Supplementary Data).

Regarding the comparative analysis of fat-free mass index and fat mass index values, all patients were included (Table 5, Supplementary Figure S2, Supplementary Data).

To evaluate the association of clinical and demographic parameters with severe postoperative complications, a Firth penalized logistic regression analysis was performed (Table 6). In the univariate analysis, both “BCC out of green” (OR 3.098, 95% CI 1.025–11.104, *p* = 0.045) and ECOG performance status (OR 1.911, 95% CI 1.046–3.694, *p* = 0.035) were significantly associated with the outcome. In contrast, ASA physical status (*p* = 0.965) and age (*p* = 0.698) showed no significant correlation in the univariate model (Table 6).

In the multivariable analysis, both variables remained independently associated with severe postoperative complications: ‘BCC out of green’ was associated with higher odds (OR 3.321, 95% CI 1.039–12.870, *p* = 0.043), and a higher ECOG score was likewise associated with higher odds of severe complications (OR 2.071, 95% CI 1.079–4.244, *p* = 0.028). Neither ASA status (*p* = 0.223) nor age (*p* = 0.905) reached statistical significance in the multivariate model (Table 6).

A detailed comparative analysis of patient demographics, anthropometric, laboratory and bioelectrical impedance variables, as well as surgical procedures of patients with and without major complications, is illustrated in Supplementary Table S1.

## 4. Discussion

Bioelectrical impedance analysis (BIA) has gained increasing relevance as an objective, bedside-accessible method to evaluate nutritional status, hydration, and cellular integrity in surgical patients [6,18]. In this retrospective cohort of 73 patients undergoing major hepatopancreaticobiliary (HPB) surgery, BIA-derived variables revealed substantial preoperative functional impairment compared with healthy, age- and BMI-matched reference populations. Specifically, when contextualized against the normative datasets by Peine et al. and Piccoli et al., 95% of our patients demonstrated strong deviations across multiple BIA domains; i.e., most consistently a reduced reactance/height (Xc/H), phase angle (PA), and fat-free mass index (FFMI), but elevated resistance/height (R/H) and fat mass index (FMI) [6,18]. The results highlight a significant clinical issue: a high proportion of patients with HPB malignancies present preoperatively with reduced muscle mass, nutritional status and indicators of reduced cell health, even though their BMI and albumin levels often fall within normal ranges. This underscores a limitation of traditional markers that has been noted in previous nutritional studies [3].

Postoperative morbidity after major HPB surgery remains substantial in contemporary publications, including national liver surgery outcomes and large multicenter pancreatic cross-sectional studies [2,20,21]. In total, 37% of our patients developed severe postoperative complications (Clavien–Dindo ≥ 3), and 30-day mortality accounted for 7%. These findings highlight the necessity for enhanced preoperative risk assessment that goes beyond typical demographic and surgical factors [4,11,12].

While malnutrition and altered body composition are recognized determinants of adverse surgical outcomes [3,5], routine clinical assessment often underdetects these abnormalities. This is particularly evident in older patients and in those with obesity or cancer-related inflammation, where weight- or BMI-based measures may be misleading due to the masking effects of fluid retention or excess adipose tissue [11,13].

Body composition indices derived from BIA showed a heterogeneous but clinically informative pattern. Compared with Peine reference data, male patients, especially those with BMI < 25, demonstrated reduced fat-free mass index (FFMI), consistent with diminished muscle mass and metabolic reserve. Importantly, fat mass index (FMI) was elevated across many BMI categories, most notably in women with BMI ≥ 25 kg/m^2^ to <30 kg/m^2^ and in men with BMI ≥ 30 kg/m^2^ [18]. This pattern is compatible with adverse body composition phenotypes in which excess adiposity coexists with reduced fat-free mass and impaired cellular integrity, an increasingly recognized high-risk state in major abdominal and oncologic surgery [3,11,13].

Beyond mass-based estimates, impedance vector components offered the clearest and most consistent signal of cellular compromise. Resistance/body height (R/H), a parameter influenced by total body water and lean tissue quantity, was modestly elevated in male patients in the Piccoli comparison (significant in BMI 19 to <25 kg/m^2^ and 25 to <30 kg/m^2^), consistent with reduced hydration/lean mass [6]. However, the most striking and uniform abnormality was the severe reduction in reactance/height and PA across all sex- and BMI-defined variables [3,13]. Compared with the reference population, reactance/height in our cohort was substantially and significantly lower in every subgroup, and phase angle values were significantly reduced (e.g., ~3.8–4.6° in patients versus ~7–8° in the reference). Xc reflects the capacitive properties of cell membranes and body cell mass, while phase angle integrates both resistance and reactance and is widely considered a global marker of cellular integrity, membrane function, and overall physiological robustness [6,13]. The magnitude and consistency of the observed phase angle depression, therefore, suggests that many patients exhibited a “cellular frailty” signal that is not captured by BMI-based assessment. Our results are consistent with the existing literature that associates a low phase angle with negative outcomes. In a systematic review of cancer surgery, Matthews et al. reported that BIA parameters with the most consistently low PA were frequently associated with increased postoperative complications, with phase angle outperforming many derived BIA estimates [3]. Similarly, malnutrition assessed via phase angle predicted outcomes even in “low-risk” cardiac surgery cohorts. In the context of hepatobiliary and pancreatic surgery, Yasui-Yamada et al. demonstrated that low preoperative PA was associated with poorer short- and long-term postoperative prognosis. These results support the relevance of preoperative PA measurements specifically to the disease spectrum represented in our cohort [12,13]. Data specific to certain procedures also lend credibility to the idea of using PA as a biomarker during the perioperative period in pancreatic surgery. Zhou et al. reported that phase angle correlated with nutritional status and predicted postoperative complications in pancreatic head cancer patients undergoing pancreatoduodenectomy (area under the curve (AUC) 0.7) [13]. A conference report published in Clinical Nutrition ESPEN focused on pancreaticoduodenectomy and indicated that a low phase angle derived from BIA is linked to increased rates of complications within 30 days post-surgery, highlighting the importance of phase angle in the context of pancreatic cancer operations [5,17]. These studies collectively support our conclusion that notably low phase angle and Xc/height indicate clinically significant preoperative vulnerability in patients undergoing major abdominal surgeries, including those involving HPB procedures [3,13].

Importantly, the multivariable Firth logistic regression analysis identified BCC classification outside the green reference zone (“BCC out of green”) as being independently associated with severe postoperative complications after adjustment for age, ASA physical status, and ECOG performance status. Patients classified as “BCC out of green” had higher odds of severe postoperative complications (OR 3.321, 95% CI 1.039–12.870, *p* = 0.043). This odds ratio should be interpreted as an increase in the odds of severe postoperative complications rather than as a threefold increase in risk. Moreover, the relatively wide confidence interval indicates considerable uncertainty regarding the magnitude of this association and limits the precision of the effect estimate. Therefore, the observed association should not be interpreted as evidence of a causal relationship or as a precise estimate of individual postoperative risk.

Higher ECOG performance status was likewise independently associated with severe postoperative complications (OR 2.071, 95% CI 1.079–4.244, *p* = 0.028), whereas ASA physical status and age were not statistically significantly associated with the outcome in this model. The absence of statistical significance for these variables should, however, not be interpreted as evidence of an absence of prognostic relevance outside the present cohort.

Moreover, BCC may provide a more integrated assessment of body composition abnormalities than phase angle alone, as phase angle can be influenced by several preanalytical and environmental factors. While phase angle is widely recognized as a global marker of cellular integrity, its raw BIA-derived nature renders it susceptible to a range of preanalytical and environmental confounders. These include hydration status, recent food and fluid intake, body position, skin temperature, physical activity prior to measurement, and the conductance properties of the examination surface. In a clinical setting, particularly in the perioperative context, where patients frequently present with altered fluid balance, inflammation-driven shifts in body water distribution, or impaired fasting compliance, these sources of variability may limit the utility of phase angle as a standalone assessment parameter [22,23,24,25,26].

Taken together, these findings suggest that BCC may provide complementary information regarding preoperative physiological and body composition abnormalities that are associated with severe postoperative morbidity. Given the retrospective observational design and the limited precision of the BCC estimate, these findings require confirmation in larger prospective cohorts before BCC can be considered an established tool for postoperative risk prediction or used to guide therapeutic interventions.

Our biochemical data support the role of prealbumin as a sensitive marker of protein-energy malnutrition: the median prealbumin serum level was ~19.8 mg/dL. In total, 52% of the patients were detected below our laboratory threshold of 20 mg/dL. Evidence has shown that low levels of prealbumin in the blood are linked to infections after surgery or negative outcomes in both cardiac and gastrointestinal procedures, highlighting its importance as an additional serum biomarker in clinical settings [8,9,10]. In this context, it is notable that albumin serum values were largely within normal ranges (median 4.0 g/dL), reinforcing that albumin, due to its long half-life and sensitivity to inflammation and fluid shifts, may fail to identify high-risk nutritional phenotypes. This limitation is consistent with longstanding evidence and guideline statements that emphasize screening and targeted nutritional intervention rather than relying on albumin alone [5,6,9].

Taken together, these results argue for a multimodal concept of preoperative nutritional risk assessment in HPB surgery that integrates screening tools (NRS) [7], biochemical markers (prealbumin) [8,9], and objective body composition/cellular integrity metrics (PA, Xc/H) [3,17]. From a practical standpoint, our findings support the routine integration of BIA into preoperative pathways for major HPB resections. BIA is non-invasive, rapid, repeatable, radiation-free, and yields a set of complementary variables that can identify high-risk phenotypes even in patients with apparently “normal” values for BMI and albumin. Reference-based interpretation using Peine for body composition and Piccoli for vector parameters can contextualize individual results and strengthen clinical decision-making [6,18]. Moreover, targeted nutritional and functional interventions could be considered for patients with severely depressed PA/Xc/H or elevated NRS since prehabilitation has shown promise in optimizing outcomes in pancreatic cancer surgery [4,13]. The ESPEN surgery guideline framework further supports nutritional therapy for patients at risk, integrated within ERAS (Enhanced Recovery After Surgery)-based perioperative care [5,7].

When interpreting these results, it is crucial to position BIA within the broader context of body composition profiling. While CT and MRI remain the gold standards for anatomical muscle mass quantification, they require specialized software, involve radiation exposure (in the case of CT), and are not dynamically repeatable at the bedside. Similarly, simple anthropometric indicators like calf circumference measurements offer bedside accessibility for evaluating muscle mass, while functional assessments such as handgrip strength provide essential insights into muscle performance; however, these standalone metrics remain limited in capturing dynamic cellular shifts or complex multi-compartment body composition changes. BIA offers a rapid and cost-effective approach that adds unique functional data regarding cellular integrity. However, it must be acknowledged that mass-based BIA estimates can overestimate actual skeletal muscle mass compared to reference imaging, particularly in patients with altered hydration states or systemic cancer-related inflammation. Consequently, BIA should be regarded as a complementary functional screening tool rather than a direct replacement for cross-sectional imaging [14,15,16].

In summary, this cohort demonstrates profound preoperative abnormalities in BIA-derived cellular integrity markers, especially BCC, PA and Xc, alongside a strong association between nutritional risk screening and severe postoperative complications. Our results reinforce that physiological reserve is a key determinant of outcome in major HPB surgery. These findings suggest that BIA may provide complementary information during preoperative assessment; its potential role in guiding targeted prehabilitation should be evaluated prospectively.

## 5. Conclusions

The combination of BIA, particularly BCC, PA and Xc/H, NRS screening, and prealbumin evaluation offers a practical approach to identifying patients at nutritional risk and diminished physiological reserve that might otherwise go unnoticed in candidates for HPB surgery. Considering the consistently high rates of complications associated with major liver and pancreatic surgeries, systematically pinpointing patients at high risk could facilitate targeted nutritional and functional enhancement through prehabilitation and better perioperative management [4,7,20,21]. Future research should investigate whether improvements in BIA-derived cellular markers, driven by interventions, lead to lower complication rates and enhanced recovery in major HPB surgery.

## 6. Limitations

This study has several limitations. As a single-center retrospective analysis, the generalizability of the findings and the ability to conduct comprehensive multivariate modelling are limited. The use of published healthy reference populations introduces potential heterogeneity due to demographic and methodological differences, and the FFMI and FMI reference data from Peine lacked stratum-specific sample sizes, preventing formal hypothesis testing.

In addition, although BCC status remained statistically associated with severe postoperative complications in the multivariable model, the wide 95% confidence interval around the corresponding odds ratio indicates limited precision of this estimate. Consequently, the magnitude of the observed association should be interpreted cautiously and requires validation in larger independent cohorts.

Furthermore, standardized prospective CT-based body composition analysis (e.g., at the L3 vertebral level) was not available for all patients, precluding a direct within-subject comparison between BIA and reference imaging. In addition, clinically relevant edema was not systematically graded in this retrospective dataset. Because fluid overload can confound impedance-derived estimates of body composition and may lead to an overestimation of skeletal muscle mass compared with CT-based assessment, our findings should be interpreted with caution. This limitation underscores our methodological focus on vector-based parameters, such as phase angle and the body composition chart (BCC), which are considered more robust indicators of cellular integrity under varying hydration conditions. Therefore, BIA should be regarded as a complementary functional assessment tool rather than an interchangeable replacement for cross-sectional imaging.

In addition, malnutrition was defined using a pragmatic combination of prealbumin, BIA findings and clinical assessment (NRS score), rather than established diagnostic systems. Larger prospective cohorts will be needed to evaluate whether prehabilitation strategies, such as immunonutrition or anabolic interventions, can improve BIA metrics and clinical outcomes.

## Figures and Tables

**Table 1:** Patient characteristics.

Outcomes	Data
Number of patients (*N*)	73
Demographic and anthropometric variables	
Age (years, IQR)	70 (61–78)
Female (*n*, %)	26 (36%)
Height (cm)	170.5 ± 8.8
BMI (kg/m^2^)	25.1 (22.6–27.4)
ASA(*n*, %)	
1	3 (4%)
2	13 (18%)
3	57 (78%)
ECOG (*n*, %)	
0	30 (41%)
1	31 (42%)
2	10 (14%)
3	2 (3%)
NRS (median)	2 (1–4)
Laboratory variables	
Hemoglobin (g/dL)	12.5 (11.4–13.6)
Albumin (g/dL)	4.0 (3.7–4.3)
Prealbumin (mg/dL)	19.8 (14.9–24.8)
Bioelectrical impedance variables	
Resistance (Ω)	541.6 (501.0–623.2)
Reactance (Ω)	41.5 (34.8–49.9)
Phase Angle (°)	4.3 (3.6–4.8)
FFMI (kg/m^2^)	18.5 (15.5–20.1)
FMI (kg/m^2^)	8.5 (6.6–10.9)
Type of surgery	
Pancreaticoduodenectomy (*n*, %)	45 (62%)
Distal pancreatectomy (*n*, %)	10 (14%)
Total pancreatectomy (*n*, %)	5 (7%)
Right hemihepatectomy (*n*, %)	5 (7%)
Other (*n*, %)	8 (10%)
Postoperative variables	
Clavien–Dindo ≥ 3 (*n*, %)	27 (37%)
30 d mortality (*n*, %)	5 (7%)
LOS (days)	15 (11–28)
Readmission within 30 days	4 (5%)
Values are presented as *n* (%), mean ± SD, or median (IQR), as appropriate	

IQR = interquartile range, BMI = body mass index, FFMI = fat-free mass index, FMI = fat mass index, ASA = American Society of Anesthesiologists Physical Status Classification System, ECOG = Eastern Cooperative Oncology Group Performance Status, LOS = length of stay, NRS = nutritional risk screening.

**Table 2:** Spectrum and severity of postoperative morbidity graded by the Clavien–Dindo classification system.

Complications	Data
None (*n*, %)	36 (49%)
Clavien–Dindo grade 1 and 2	10 (14%)
Surgical site infection Grade 1 (*n*, %)	4 (6%)
Delayed gastric emptying (*n*, %)	2 (3%)
Others (*n* %)	4 (6%)
Clavien–Dindo grade 3	13 (18%)
Intraabdominal retention requiring endoscopic intervention (*n*, %)	5 (7%)
Postoperative pancreatic fistula Type B/C (*n*, %)	5 (7%)
Abdominal dehiscence and major surgical site infections (*n*, %)	3 (4%)
Clavien–Dindo grade 4	9 (12%)
Bleeding with operative intervention (*n*, %)	4 (6%)
Insufficiency of anastomosis with postoperative pancreatic fistula with operative intervention (*n*, %)	4 (6%)
Others (*n*, %)	1 (1%)
Clavien–Dindo grade 5	5 (7%)
Cardiovascular and vascular ischemic events (*n*, %)	3 (4%)
Systemic organ failure and unspecified causes (*n*, %)	2 (3%)
Values are presented as *n* (%) as appropriate	

**Table 3:** Body height-normalized resistance, reactance, phase angle, fat-free mass index (FFMI) and fat mass index (FMI) values of the study cohort.

Outcomes	Female	Male
Resistance/Height (Ω/cm)		
All	3.76 ± 0.74	3.07 ± 0.57
BMI < 25	3.98 ± 0.71	3.38 ± 0.67
BMI ≥ 25 < 30	3.50 ± 0.49	2.87 ± 0.36
BMI ≥ 30	2.79 ± 0.99	2.78 ± 0.39
Reactance/Height (Ω/cm)		
All	0.26 ± 0.07	0.24 ± 0.06
BMI < 25	0.27 ± 0.07	0.25 ± 0.06
BMI ≥ 25 < 30	0.24 ± 0.08	0.23 ± 0.05
BMI ≥ 30	0.23 ± 0.11	0.22 ± 0.07
Phase angle (°)		
All	3.85 ± 0.87	4.41 ± 0.73
BMI < 25	3.78 ± 0.88	4.28 ± 0.67
BMI ≥ 25 < 30	3.83 ± 0.94	4.55 ± 0.77
BMI ≥ 30	4.50 ± 0.71	4.32 ± 0.75
FFMI (kg/m^2^)		
BMI < 25	14.42 ± 1.56	17.15 ± 2.13
BMI ≥ 25 < 30	17.33 ± 2.20	20.05 ± 2.28
BMI ≥ 30	23.85 ± 5.30	21.55 ± 2.34
FMI (kg/m^2^)		
BMI < 25	7.31 ± 1.96	6.37 ± 1.86
BMI ≥ 25 < 30	14.91 ± 10.14	8.22 ± 2.20
BMI ≥ 30	17.45 ± 8.13	12.98 ± 1.97

BMI = body mass index, FFMI = fat-free mass index, FMI = fat mass index.

**Table 4:** Comparative analysis of resistance, reactance and phase angle values between the study cohort and an age- and BMI-matched healthy control population.

Outcomes		Female				Male		
	Patient	Control	Glass’ Δ	*p*-Value	Patient	Control	Glass’ Δ	*p*-Value
Resistance/Height (Ω/cm)								
BMI 19 – < 25	3.94 ± 0.72	3.83 ± 0.45	0.227	0.623	3.32 ± 0.62	2.92 ± 0.34	1.170	0.016
BMI 25 – < 30	3.50 ± 0.49	3.55 ± 0.36	−0.138	0.798	2.87 ± 0.36	2.66 ± 0.29	0.707	0.014
BMI 30 – < 35	N/A	N/A	N/A	N/A	2.78 ± 0.38	2.42 ± 0.25	1.391	0.074
Reactance/Height (Ω/cm)								
BMI 19 – < 25	0.28 ± 0.07	0.48 ± 0.07	−2.847	<0.001	0.25 ± 0.05	0.40 ± 0.06	−2.530	<0.001
BMI 25 – < 30	0.24 ± 0.08	0.45± 0.06	−3.186	0.001	0.23 ± 0.05	0.37 ± 0.06	−2.361	<0.001
BMI 30 – < 35	N/A	N/A	N/A	N/A	0.22 ± 0.07	0.33 ± 0.05	−2.130	0.019
Phase Angle (°)								
BMI 19 – < 25	3.93 ± 0.88	7.14 ± 0.87	−3.699	<0.001	4.26 ± 0.69	7.84 ± 1.01	−3.536	<0.001
BMI 25 – < 30	3.83 ± 0.94	7.21 ± 0.85	−3.960	<0.001	4.55 ± 0.77	7.82 ± 1.04	−3.159	<0.001
BMI 30 – <35	N/A	N/A	N/A	N/A	4.32 ± 0.75	7.78 ± 1.03	−3.360	<0.001

BMI = body mass index, N/A = not available.

**Table 5:** Comparative analysis of fat-free mass index and fat mass index between the study cohort and an age- and BMI-matched healthy control population.

Outcomes	Female			Male		
	Patient	Control	Glass’ Δ	Patient	Control	Glass’ Δ
FFMI (kg/m^2^)						
BMI < 25	14.42 ± 1.56	15.63 ± 0.92	−1.318	17.15 ± 2.13	18.81 ± 1.03	−1.609
BMI ≥ 25 < 30	17.33 ± 2.20	16.88 ± 0.93	0.482	20.05 ± 2.28	20.08 ± 1.03	−0.029
BMI ≥ 30	23.85 ± 5.30	18.48 ± 1.27	4.228	21.55 ± 2.34	22.09 ± 1.24	−0.435
FMI (kg/m^2^)						
BMI < 25	7.31 ± 1.96	6.55 ± 1.39	0.548	6.37 ± 1.86	4.23 ± 1.22	1.753
BMI ≥ 25 < 30	14.91 ± 10.14	10.30 ± 1.34	3.438	8.22 ± 2.20	7.08 ± 1.28	0.893
BMI ≥ 30	17.45 ± 8.13	15.04 ± 2.65	0.909	12.98 ± 1.97	10.79 ± 1.99	1.102

BMI = body mass index, FFMI = fat-free mass index, FMI = fat mass index.

**Table 6:** Firth logistic regression analysis for parameters associated with severe postoperative complications (Clavien–Dindo ≥ 3).

	Univariate			Multivariate		
Parameter	OR	CI (95%)	*p*-Value	OR	CI (95%)	*p*-Value
BCC out of green	3.098	1.025–11.104	0.045	3.321	1.039–12.87	0.043
ECOG	1.911	1.046–3.694	0.035	2.071	1.079–4.244	0.028
ASA	0.981	0.417–2.466	0.965	0.532	0.192–1.494	0.223
Age	1.007	0.971–1.048	0.698	1.002	0.963–1.045	0.905

ASA = American Society of Anesthesiologists Physical Status Classification System, ECOG = Eastern Cooperative Oncology Group Performance Status, OR = odds ratio, RD = risk difference, CI (95%) = confidence interval within 95%, LOS = length of stay.

## Data Availability

The original contributions presented in the study are included in the article/Supplementary Material; further inquiries can be directed to the corresponding author, Clemens Schmutzhart (c.schmutzhart@salk.at).

## Supplementary Materials

The following supporting information can be downloaded at: https://www.mdpi.com/article/10.3390/diagnostics16172792/s1, Figure S1: Comparison of bioelectrical impedance parameters between patients and controls, stratified by sex and BMI: (a) resistance/height; (b) reactance/height; (c) phase angle; Figure S2: Comparison of body composition indices between patients and controls, stratified by sex and BMI: (a) fat-free mass index (FFMI); (b) fat mass index (FMI); Table S1: Patient demographics, anthropometric, laboratory, bioelectrical impedance variables, and surgical characteristics stratified by major postoperative complications.
