# Supplementary material for: Preoperative Bioelectrical Impedance Analysis Identifies Altered Body Composition, Which Is Associated with Severe Postoperative Complications in Major Hepatopancreaticobiliary Surgery
Source: Diagnostics (Basel). 2026 Aug 30;16(17):2792. doi: 10.3390/diagnostics16172792 (PMC13565584; doi:10.3390/diagnostics16172792)
Supplement: Supplementary file 1 [file diagnostics-16-02792-s001.zip › diagnostics-4443035-supplementary.pdf]

Supplementary Materials

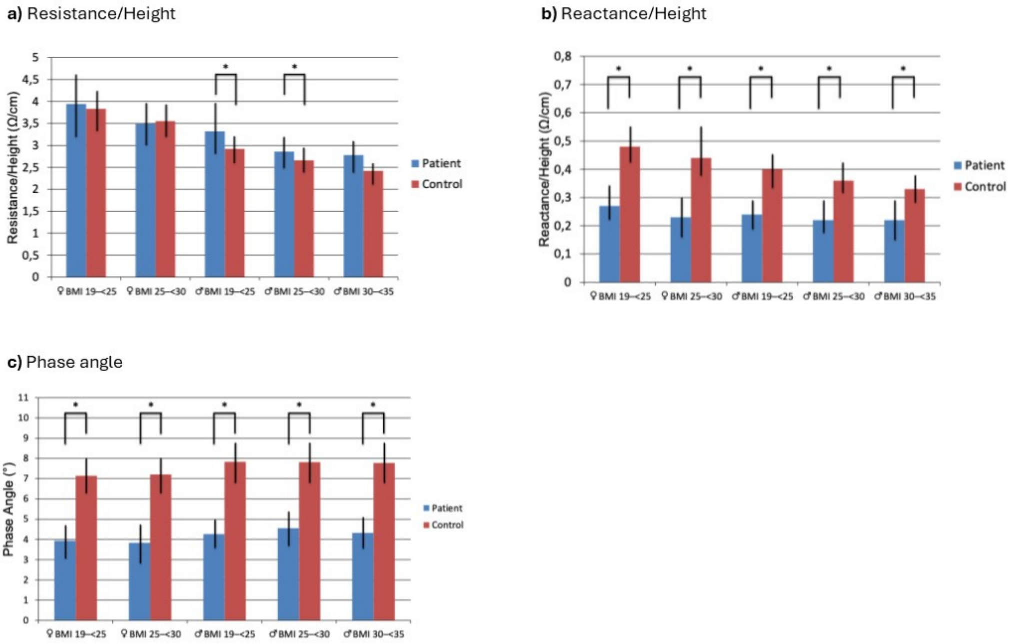

Figure S1. Comparison of bioelectrical impedance parameters between patients and controls, stratified by sex and BMI: (a) resistance/height; (b) reactance/height; (c) phase angle. Data are presented as mean ± SD; *$p < 0.05$; ♀ = female, ♂ = male.

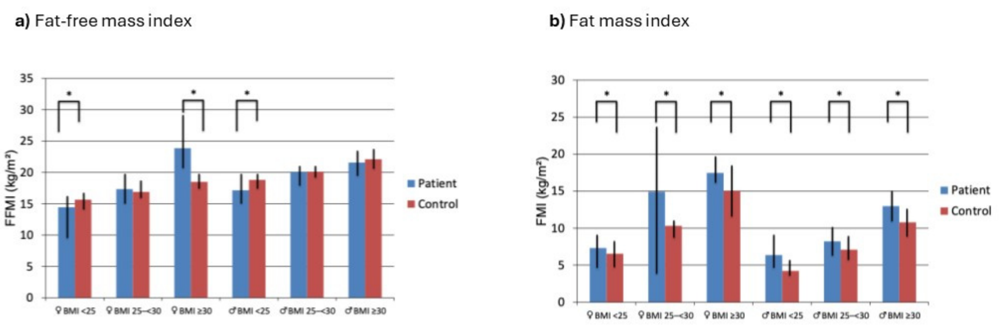

Figure S2. Comparison of body composition indices between patients and controls, stratified by sex and BMI: (a) fat-free mass index (FFMI); (b) fat mass index (FMI). Data are presented as mean ± SD; *$p < 0.05$; ♀ = female, ♂ = male.

Supplementary Text for Figures S1 and S2

Comparative analysis of resistance, reactance, phase angle, fat-free mass index and fat mass index

In female patients, the R-to-height ratio was measured at 3.94 ± 0.72 Ω/cm, compared to 3.83 ± 0.45 Ω/cm in the reference cohort for a BMI range of 19 to <25 ($p = 0.623$), and 3.50 ± 0.49 Ω/cm versus 3.55 ± 0.36 Ω/cm for a BMI range of 25 to <30 ($p = 0.798$). The reactance-to-height ratio was 0.28 ± 0.07 Ω/cm, compared to 0.48 ± 0.07 Ω/cm for a BMI range of 19 to <25 ($p < 0.001$), and 0.24 ± 0.08 Ω/cm versus 0.45 ± 0.06 Ω/cm for a BMI range of 25 to <30 ($p = 0.001$). Phase angle values were recorded at 3.93 ± 0.88° compared to 7.14 ± 0.87° for a BMI range of 19 to <25 ($p < 0.001$), and 3.83 ± 0.94° versus 7.21 ± 0.85° for a BMI range of 25 to <30 ($p < 0.001$) (Table 4, Figure S1a–c).

In male patients, the R-to-height ratio was 3.32 ± 0.62 Ω/cm, compared to 2.92 ± 0.34 Ω/cm in the reference cohort for a BMI of 19 to <25 ($p = 0.016$), 2.87 ± 0.36 Ω/cm versus 2.66 ± 0.29 Ω/cm for a BMI of 25 to <30 ($p = 0.014$), and 2.78 ± 0.38 Ω/cm versus 2.42 ± 0.25 Ω/cm for a BMI of 30 to <35 ($p = 0.074$). The Xc-to-height ratio was measured at 0.25 ± 0.05 Ω/cm, compared to 0.40 ± 0.06 Ω/cm for a BMI of 19 to <25 ($p < 0.001$), 0.23 ± 0.05 Ω/cm versus 0.37 ± 0.06 Ω/cm for a BMI of 25 to <30 ($p < 0.001$), and 0.22 ± 0.07 Ω/cm versus 0.33 ± 0.05 Ω/cm for a BMI of 30 to <35 ($p = 0.019$). PA values were 4.26 ± 0.69° compared to 7.84 ± 1.01° for a BMI of 19 to <25 ($p < 0.001$), 4.55 ± 0.77° versus 7.82 ± 1.04° for a BMI of 25 to <30 ($p < 0.001$), and 4.32 ± 0.75° versus 7.78 ± 1.03° for a BMI of 30 to <35 ($p < 0.001$) (Table 4, Figure S1a–c).

For FFMI, female patients with a BMI < 25 showed a mean value of 14.42 ± 1.56 kg/m^2 compared with 15.63 ± 0.92 kg/m^2 in the reference cohort, corresponding to a Glass' Δ of −1.318. In the BMI ≥ 25 to < 30 group, FFMI measured 17.33 ± 2.20 kg/m^2 in patients versus 16.88 ± 0.93 kg/m^2 in controls (Glass' Δ = 0.482). For patients with BMI ≥ 30, mean FFMI was 23.85 ± 5.30 kg/m^2 compared with 18.48 ± 1.27 kg/m^2 in the reference cohort, yielding a Glass' Δ of 4.228 (Table 5, Figure S2a–b).

In male patients, FFMI was 17.15 ± 2.13 kg/m^2 for BMI < 25 compared with 18.81 ± 1.03 kg/m^2 in controls (Glass' Δ = −1.609). In the BMI ≥ 25 to < 30 category, values were 20.05 ± 2.28 kg/m^2 in patients and 20.08 ± 1.03 in the reference cohort (Glass' Δ = −0.029). For BMI ≥ 30, FFMI measured 21.55 ± 2.34 kg/m^2 in patients compared with 22.09 ± 1.24 kg/m^2 in controls, corresponding to a Glass' Δ of −0.435. FMI in female patients with BMI < 25 demonstrated a mean value of 7.31 ± 1.96 kg/m^2 compared with 6.55 ± 1.39 kg/m^2 in the reference cohort, with a Glass' Δ of 0.548. In the BMI ≥ 25 to < 30 group, FMI increased to 14.91 ± 10.14 kg/m^2 in patients versus 10.30 ± 1.34 in controls (Glass' Δ = 3.438). For BMI ≥ 30, FMI was 17.45 ± 8.13 kg/m^2 compared with 15.04 ± 2.65 kg/m^2 in the reference cohort, yielding a Glass' Δ of 0.909. In male patients, FMI values were 6.37 ± 1.86 kg/m^2 for BMI < 25 compared with 4.23 ± 1.22 kg/m^2 in controls (Glass' Δ = 1.753). In the BMI ≥ 25 to < 30 category, FMI measured 8.22 ± 2.20 kg/m^2 in patients versus 7.08 ± 1.28 kg/m^2 in the reference cohort (Glass' Δ = 0.893). For BMI ≥ 30, FMI was 12.98 ± 1.97 kg/m^2 compared with 10.79 ± 1.99 kg/m^2 in controls, corresponding to a Glass' Δ of 1.102 (Table 5, Figure S2a–b).

Table S1. Patient demographics, anthropometric, laboratory, bioelectrical impedance variables, and surgical characteristics stratified by major postoperative complications.

Outcomes	Major complications	No major complications	*p*-Value
Number of patients (n, %)	27 (37%)	46 (63%)	
**Demographic and anthropometric variables**			
Age (years, IQR)	70 (58–78)	69.5 (61–77)	0.837
Female (n, %)	9 (33%)	17 (37%)	0.755
Height (cm)	171.6 ± 8.7	170.1 ± 8.8	0.431
BMI (kg/m^2)	26.60 (23.1–29.2)	24.6 (22.2–27.0)	0.049
**ASA (n, %)**			
1	1 (4%)	2 (4%)	1
2	5 (19%)	8 (17%)	1
3	21 (78%)	36 (78%)	0.962
**ECOG (n, %)**			
<2	20 (74%)	41 (89%)	0.112
≥2	7 (26%)	5 (11%)	0.112
BCC out of green	22 (82%)	28 (61%)	0.051
**Laboratory variables**			
Hemoglobin (g/dL)	13.10 (11.6–13.8)	12.4 (11.1–13.5)	0.401
Albumin (g/dL)	3.9 (3.5–4.3)	4.0 (3.6–4.3)	0.651
Prealbumin (mg/dL)	19.1 (14.5–27.0)	20.4 (15.0–24.5)	0.916
**Bioelectrical impedance variables**			
Resistance (Ω)	529.5 (478.1–636.9)	545.8 (513.4–623.1)	0.426
Reactance (Ω)	40.5 (32.7–46.9)	42.3 (36.1–50.0)	0.630
Phase Angle (°)	4.5 (3.6–4.7)	4.15 (3.6–4.8)	0.668
FFMI (kg/m^2)	18.5 (15.1–20.7)	17.5 (15.5–19.4)	0.253
FMI (kg/m^2)	8.5 (6.5–11.7)	8.1 (6.4–9.1)	0.263
**Type of surgery**			
Pancreaticoduodenectomy (n, %)	15 (55%)	30 (65%)	0.412
Distal pancreatectomy (n, %)	6 (22%)	4 (9%)	0.158
Total pancreatectomy (n, %)	2 (7%)	3 (7%)	1
Right hemihepatectomy (n, %)	2 (7%)	3 (7%)	1
Other (n, %)	2 (7%)	6 (13%)	0.702

Values are presented as n (%), mean ± SD, or median (IQR), as appropriate. IQR = interquartile range; BMI = body mass index; FFMI = fat-free mass index; FMI = fat mass index; ASA = American Society of Anesthesiologists Physical Status Classification System; ECOG = Eastern Cooperative Oncology Group Performance Status; BCC = Body Composition Chart.

Supplementary Text for Table S1

In the major complications group, the median age was 70 years (IQR 58–78) and 9 patients (33%) were female, while the no major complications group had a median age of 69.5 years (IQR 61–77) with 17 female patients (37%). The mean body height was 171.6 cm with a standard deviation of 8.7 cm in the major complications group, compared to 170.1 cm with a standard deviation of 8.8 cm in the group without major complications. The median body mass index was 26.60 kg/m² (IQR 23.1–29.2) in patients with major complications and 24.6 kg/m² (IQR 22.2–27.0) in those without (Table S1).

With regard to preoperative physical status in the major complications group, 1 patient (4%) was classified as ASA 1, 5 patients (19%) as ASA 2, and 21 patients (78%) as ASA 3; in the no major complications group, 2 patients (4%) were classified as ASA 1, 8 patients (17%) as ASA 2, and 36 patients (78%) as ASA 3.

Regarding ECOG performance status, 20 patients (74%) in the major complications group had an ECOG performance status <2, while 7 patients (26%) had an ECOG performance status ≥2. In the group without major complications, 41 patients (89%) had an ECOG performance status <2 and 5 patients (11%) had an ECOG performance status ≥2. Additionally, "BCC out of green" was observed in 22 patients (82%) with major complications, compared with 28 patients (61%) in the group without major complications (Table S1).

Bioelectrical impedance analysis in the major complications group revealed a median R of 529.5 Ω (IQR 478.1–636.9), a median Xc of 40.5 Ω (IQR 32.7–46.9), and a median PA of 4.5° (IQR 3.6–4.7), with an FFMI of 18.5 kg/m² (IQR 15.1–20.7) and an FMI of 8.5 kg/m² (IQR 6.5–11.7). In comparison, the no major complications group showed a median R of 545.8 Ω (IQR 513.4–623.1), a median Xc of 42.3 Ω (IQR 36.1–50.0), a median PA of 4.15° (IQR 3.6–4.8), an FFMI of 17.5 kg/m² (IQR 15.5–19.4), and an FMI of 8.1 kg/m² (IQR 6.4–9.1) (Table S1).

For hematological variables, patients with major complications presented with a median hemoglobin concentration of 13.10 g/dL (IQR 11.6–13.8), a median serum albumin level of 3.9 g/dL (IQR 3.5–4.3), and a median prealbumin concentration of 19.1 mg/dL (IQR 14.5–27.0). In the group without major complications, the median hemoglobin concentration was 12.4 g/dL (IQR 11.1–13.5), the median serum albumin level was 4.0 g/dL (IQR 3.6–4.3), and the median prealbumin concentration was 20.4 mg/dL (IQR 15.0–24.5) (Table S1).

Regarding surgical interventions, patients with major complications underwent pancreaticoduodenectomy in 15 cases (55%), distal pancreatectomy in 6 cases (22%), total pancreatectomy in 2 cases (7%), right hemihepatectomy in 2 cases (7%), and other procedures in 2 cases (7%). In the no major complications group, 30 patients (65%) underwent pancreaticoduodenectomy, 4 patients (9%) underwent distal pancreatectomy, 3 patients (7%) underwent total pancreatectomy, 3 patients (7%) underwent right hemihepatectomy, and 6 patients (13%) underwent other procedures (Table S1).
